# Detection of Lamin A in mesenteric neurons: Accurate diagnostic method in patients with sporadic Alzheimer's Disease

**DOI:** 10.1002/alz.087988

**Published:** 2025-01-09

**Authors:** Laura Gil Alberdi, Carmen Guerrero, Ana Belen Rebolledo

**Affiliations:** ^1^ Chromatrix, Madrid, Madrid Spain; ^2^ HOSPITAL UNIVERSITARIO FUNDACION ALCORCON, MADRID Spain

## Abstract

**Background:**

Lamin A is barely expressed in human brain neurons or in murine models such as mice and rats. However, in Alheimer´s disease (AD) brains, neurons in the hippocampus and entorhinal cortex abnormally express lamina A from the initial stages of the disease, being a biomarker together with phosphorylated Tau of the nuclear pathology of AD.

Constipation and mesenteric neuronal loss are related to aging and neurodegenerative diseases such as AD. It has been suggested that tau could be involved in enteric dysfunction, since it is expressed in enteric neurons and its presence has been reported in colon samples from AD patients. There is a bidirectional communication mechanism between the brain and the gastrointestinal system in which both organs influence the function of the other, suggesting that disease in either organ can affect the function of the other.

The SPAM mouse model (expresses human tau with aggregating mutations S320F and P301S) in addition to presenting the neuronal cytoskeletal pathology of tau that manifests in AD (Braak V‐VI), expresses mutant phosphorylated tau in the cytoplasm of neurons of the myenteric plexus causing enteric neuronal death and functional impairment. SPAM tau mice also exhibit accumulation of phosphorylated tau in enteric neurons leading to significant neuronal loss and an early lethal intestinal phenotype.

**Method:**

Paraffin sections obtained from colon biopsies from AD patients diagnosed post‐mortem as well as from healthy controls were processed for immunohistochemistry. The presence of the Lamin A in the nucleus of mesenteric neurons was analysed.

**Result:**

The analysis by Immunohistochemistry showed the low nuclear expression of the biomarker Lamin A in the mesenteric neurons of the healthy samples and the overexpression of the biomarker in the colon samples of those diagnosed with AD.

**Conclusion:**

The results presented in this study show that Lamin A is clearly overexpressed in enteric neurons of patients with AD, unlike what happens in the enteric neurons of controls, which would indicate that neuronal nuclear pathology manifests in both systems, brain and colon, of patients with AD. This associated neurological implication suggests the possibility of using lamin A for therapeutic detection of AD.

